# The Association between Vitamin D Status and the Impact of Metformin on Hypothalamic–Pituitary–Thyroid Axis Activity in Women with Subclinical Hypothyroidism

**DOI:** 10.3390/pharmaceutics16081093

**Published:** 2024-08-20

**Authors:** Robert Krysiak, Karolina Kowalcze, Witold Szkróbka, Bogusław Okopień

**Affiliations:** 1 Department of Internal Medicine and Clinical Pharmacology, Medical University of Silesia, Medyków 18, 40-752 Katowice, Poland; wszkrobka@sum.edu.pl (W.S.);; 2 Department of Pediatrics in Bytom, Faculty of Health Sciences in Katowice, Medical University of Silesia, Stefana Batorego 15, 41-902 Bytom, Poland; kkowalcze@sum.edu.pl; 3 Department of Pathophysiology, Faculty of Medicine, Academy of Silesia, Rolna 43, 40-555 Katowice, Poland

**Keywords:** insulin resistance, metformin, thyrotrope secretory function, vitamin D status, women

## Abstract

Metformin inhibits enhanced secretion of anterior pituitary hormones. Its impact on prolactin and gonadotropin concentrations is absent in individuals with hypovitaminosis D. The aim of this prospective cohort study was to investigate whether vitamin D status determines the effect of metformin on hypothalamic–pituitary–thyroid axis activity in levothyroxine-naïve women. The study included three groups of women of reproductive age with subclinical non-autoimmune hypothyroidism, which were matched for age, thyroid-stimulating hormone (TSH) concentration, and insulin sensitivity: untreated women with vitamin D deficiency/insufficiency (group A), women effectively supplemented with exogenous calciferol (group B), and untreated women with normal 25-hydroxyvitamin D concentrations (25OHD) (group C). Owing to concomitant type 2 diabetes or prediabetes, all subjects were treated with metformin. Concentrations of 25OHD, TSH, total and free thyroid hormones, glucose, insulin, glycated hemoglobin (HbA_1c_), prolactin, and peripheral markers of thyroid hormone action were assayed before metformin treatment and six months later. Based on hormone concentration, structure parameters of thyroid homeostasis were calculated. Except for 25OHD concentrations, there were no between-group differences in baseline values of the measured variables. Metformin reduced glucose, the homeostatic model assessment 1 of insulin resistance ratio (HOMA1-IR), and HbA_1c_ in all study group, but these effects were less pronounced in group A than in the remaining groups. The reduction in TSH and Jostel’s index was observed only in groups B and C, and its degree correlated with baseline TSH concentrations, baseline 25OHD concentrations, and the degree of improvement in HOMA1-IR. The drug did not affect circulating levels of 25OHD, free and total thyroid hormones, prolactin, other structure parameters of thyroid homeostasis, and markers of thyroid hormone action. The obtained results allow us to conclude that low vitamin D status in young women mitigates the impact of metformin on thyrotroph secretory function.

## 1. Introduction

In spite of anatomical and functional connections with the brain, the pituitary is an organ located beyond the blood–brain barrier [1]. This fact may explain why the gland accumulates much higher quantities of metformin, the most commonly used antidiabetic agent in the world and the only American-Diabetes-Association-recommended drug for prediabetes [2,3], compared with the central nervous system, both after acute and chronic administration [4]. An important consequence of abundant pituitary accumulation is the inhibitory effect on secretory function of different populations of pituitary cells: lactotrophs [5,6,7,8], gonadotrophs [9,10,11], and thyrotrophs [12,13,14,15,16,17,18]. Metformin action on secretory function of thyrotropes was found to be determined by at least three factors. The most important one is baseline secretory function of these cells. The drug reduced thyroid-stimulating hormone (TSH) concentration only if baseline concentrations were elevated, irrespective of the reason for TSH excess: in subjects with thyroid hypofunction [12,13,14,15,16,17] and in the case of resistance to thyroid hormone [18]. In turn, no changes in TSH concentration were reported in individuals with normal TSH levels [12,13,16] and in subjects with a low concentration of this hormone resulting from hyperthyroidism [19]. This finding deserves underlining, because, unlike thyroid hormone substitution, metformin treatment does not seem to pose a risk of drug-induced thyrotoxicosis. Interestingly, similar relationships between baseline hormone concentration and the impact of treatment were also reported for prolactin and gonadotropins [6,7,9]. The second determinant of metformin action on activity of the hypothalamic–pituitary–thyroid axis is sex. The effect of metformin on TSH concentration is stronger in females than in males, which is probably a consequence of interactions between the drug and sex hormones at the level of thyrotrophs and/or hypothalamic cells producing thyrotropin-releasing hormone [20]. Thirdly, the effect may depend on the degree of metformin accumulation in the pituitary. Statistically significant changes in pituitary hormone concentrations were observed in subjects receiving high doses of this drug (2550–3000 mg per day) but not after its lower doses (1700 mg per day) [7,11]. Interestingly, despite differences in the thyroid’s secretory capacity, the impact on TSH concentration did not differ between women with hypothyroidism of autoimmune and non-autoimmune origin [21].

Recent studies of our research group showed that pituitary effects of metformin are modulated by vitamin D (calciferol) homeostasis. Subjects with low concentrations of 25-hydroxyvitamin D (25OHD) were characterized by no changes in prolactin and gonadotropin concentrations in response to metformin, though the drug was administered to women with elevated concentrations of these hormones and was able to reduce prolactin and gonadotropins in females with normal 25OHD levels [22,23]. No similar data are available for TSH. However, exogenous vitamin D was found to potentiate the inhibitory metformin action on thyrotroph secretory function in women with Hashimoto thyroiditis and high-normal TSH levels [24]. Owing to the study design, it cannot be excluded that the reduction in TSH concentration might have been caused by the improvement in thyroid function secondary to anti-inflammatory effects of exogenous vitamin D. In line with this explanation low 25OHD is frequently associated with thyroid autoimmunity, while administration of calciferol preparations reduced antibody titers [25,26]. Moreover, we cannot rule out interactions between metformin and exogenous cholecalciferol at the level of the alimentary tract. Thus, the aim of the current study was to determine whether vitamin D status determines metformin action on hypothalamic–pituitary–thyroid axis activity in individuals with non-autoimmune thyroiditis. In addition to assessment of TSH and thyroid hormone, we calculated structure parameters of thyroid homeostasis, peripheral markers of thyroid hormone action, and prolactin levels. The structure parameters of thyroid homeostasis were developed to quantify fundamental properties of the hypothalamic–pituitary–thyroid axis: thyrotropic function of the adenohypophysis (Jostel’s index), secretory capacity of the thyroid gland (structure parameter inference approach-GT (SPINA-GT)), and sum activity of peripheral deiodinases (enzymes converting thyroxine to triiodothyronine) (SPINA-GD), based on mathematical and simulative modeling of pituitary–thyroid feedback [27,28]. Plasma ferritin and osteocalcin are regarded as markers indicative of tissue thyroid hormone status and action [29]. It is well evidenced that both hepatic ferritin expression and the synthesis and secretion of osteocalcin in osteoblasts are stimulated by triiodothyronine via thyroid hormone receptors [30]. Lastly, prolactin was measured because its elevated levels are detectable in up to 40% of patients with overt primary hypothyroidism, and up to 22% of individuals with subclinical hypothyroidism [31], and possible differences in prolactin levels might have theoretically modulated the impact of metformin on thyrotrope secretory function [22]. The reason for the daily metformin dose of 3000 mg was the fact that the impact of this drug on secretory function of anterior pituitary cells was found to be dose-dependent, which suggests lower sensitivity of these cells to metformin in comparison with other target organs (the liver, gut, muscle cells, and adipose tissue) [7,11].

## 2. Materials and Methods

The protocol was approved by an institutional review board (the Bioethical Committee of the Medical University of Silesia—KNW/0022/KB/234/17; 17 October 2017), and all study procedures were performed in accordance with the Declaration of Helsinki. All participants provided written informed consent after receiving a complete description of the study. Because the participants were not randomized and received the same drug, the study did not require registration at a clinical trial registry.

### 2.1. Patients

In this single-center, cohort study, we prospectively enrolled 78 control subjects with non-autoimmune subclinical hypothyroidism, not receiving thyroid hormone substitution, and untreated type 2 diabetes or prediabetes. All were females with ages ranging between 20 and 50 years. The participants had to meet the following inclusion criteria: (a) plasma TSH concentration between 4.5 and 10.0 mU/L; (b) total thyroxine concentration between 60 and 160 nmol/L; (c) free thyroxine concentration between 10.0 and 22.0 pmol/L; (d) total triiodothyronine concentration between 1.2 and 3.2 pmol/L; (e) free triiodothyronine concentration between 2.3 and 6.7 pmol/L; (f) concentrations of thyroid peroxidase antibodies, thyroglobulin antibodies, and antibodies against TSH receptor within the normal range; and (g) no ultrasound features of thyroid autoimmunity. The widely accepted criteria were used to diagnose type 2 diabetes and prediabetes. Diabetes was diagnosed in patients with fasting plasma glucose concentration equal to 126 mg/dL or higher or 2 h post-glucose-challenge plasma glucose greater than or equal to 200 mg/dL. In turn, prediabetes was diagnosed in patients with fasting plasma glucose ranging from 100 to 125 mg/dL and/or in patients with 2-h postload plasma glucose ranging from 140 and 199 mg/dL). Only women complying with lifestyle recommendations for not less than 12 weeks were considered for enrollment. On the basis of plasma 25OHD concentrations and using exogenous calciferol supplementation, they were assigned to one of three groups. A preliminary sample size calculation showed that the estimated sample size required for a given power (80%), confidence (95%), and effect size (20% difference in TSH concentration) was 23 patients per group. Assuming possible dropouts, the number of patients in each group was increased to 26. Group A enrolled subjects with actual calciferol deficiency or insufficiency, defined as 25OHD concentrations below 75 nmol/L (30 ng/mL) [32]. For ethical reasons, individuals with 25OHD levels below 50 nmol/L (20 ng/mL) were considered eligible for enrollment only if they did not accept treatment with oral calciferol supplements. The remaining groups consisted of individuals with 25OHD concentrations between 75 and 150 nmol/L (between 30 and 60 ng/mL). Group B included females receiving, because of previous vitamin D deficiency or insufficiency, chronic (at least six-month) supplementation with stable doses of oral vitamin D preparations (50-100 μg (2000–4000 IU) daily). In turn, group C (the reference group) enrolled individuals not receiving specific oral calciferol supplements. Because of a greater number of individuals meeting the study criteria (n = 125), only some of them were recruited, and the aim of this strategy was to match the groups for age, body mass index (BMI), and TSH concentration. Matching was based on the minimum Euclidean distance rule. Owing to seasonal variation in 25OHD observed at high latitudes [33], 39 patients (14 in group A, 12 in group B, and 13 in group C) were recruited in January or February, while the remaining 39 patients (12 in group A, 14 in group B, and 13 in group C) in July or August.

We excluded women with other endocrine disorders, autoimmune or inflammatory diseases, estimated glomerular filtration rate less than 60 mL/min/1.73 m^2^, hepatic failure, malabsorption syndromes, or any other serious disorder; women receiving antidiabetic agents or other treatments (except for exogenous calciferol preparations in group B); and women who were pregnant, breastfeeding, or poorly compliant.

### 2.2. Study Design

**Figure 1:**
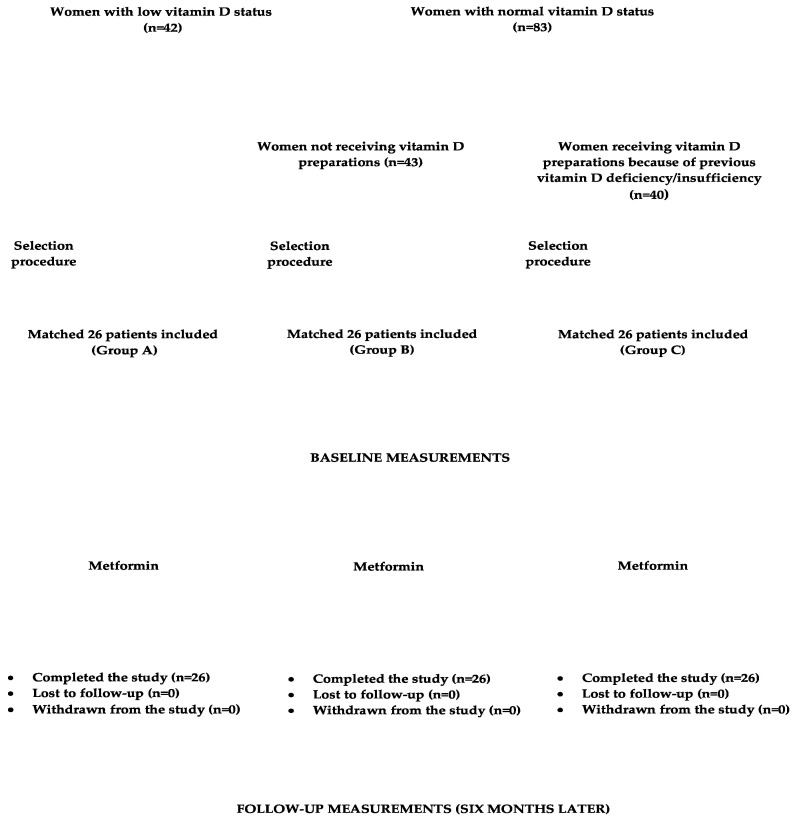
The flow chart of participants through the study.

Initial dosing for metformin was 500 mg orally twice per day. Daily dosing was then increased in 500 mg increments weekly to a target dose of 3000 mg, divided into the equal doses (1000 mg). The drug was taken 15 min prior to a meal. During the study period, non-pharmacological interventions (diet and physical activity), and, in group B, dosing of vitamin D supplements remained the same as before the study initiation. Compliance with medication regimen was assessed bimonthly using tablet counts. The daily intake of calciferol contained in food was assessed using an analysis of individual dietary questionnaires, which assessed how often and how much in the last two weeks they had consumed each of the twenty most commonly used meals of Polish cuisine. This allowed us to calculate the mean daily consumption of these meals. The obtained amounts were then multiplied by vitamin D content in each meal and summed together. The necessary data were obtained from food composition tables elaborated by the Polish National Food and Nutrition Institute in Warsaw [34]. The flow chart of participants through the study is depicted in Figure 1.

### 2.3. Laboratory Assays

All variables were determined before and after six months of metformin treatment. Venous blood samples were taken 12 h after the last meal from the antecubital vein in a temperature-controlled room (24–25 °C). Measurements were made in duplicate to ensure reproducibility. Plasma levels of glucose and whole blood content of glycated hemoglobin (HbA_1c_) were measured using the COBAS Integra 400 Plus multi-analyzer (Roche Diagnostics, Basel, Switzerland). Plasma concentrations of TSH, total thyroxine, free thyroxine, total triiodothyronine, free triiodothyronine, insulin, 25OHD, prolactin, ferritin, and 25-hydroxyvitamin D were assayed by direct chemiluminescence using acridinium ester technology (ADVIA Centaur XP Immunoassay System, Siemens Healthcare Diagnostics, Munich, Germany). Plasma concentrations of high-sensitivity C-reactive protein (hsCRP) and osteocalcin were measured by immunoassay with chemiluminescent detection (Immulite 2000XPi, Siemens Healthcare, Warsaw, Poland). The homeostatic model assessment 1 of insulin resistance ratio (HOMA1-IR) was calculated as the product of the fasting insulin level (mIU/L) and the fasting glucose level (mg/dL) divided by 405. The structure parameters of thyroid homeostasis, Jostel’s index, SPINA-GT, and SPINA-GD [28,29], were calculated using SPINA-Thyr 4.0.1 for Mac Universal software, which is available online.

### 2.4. Statistical Analysis

Prior to data analysis, all variables were subjected to log transformation to mitigate the effects of the non-normal distributions. Upon transformation, the data became normally distributed, and hence parametric statistics were used. The study groups were compared using one-way ANOVA followed by Bonferroni’s post hoc multiple comparison test. Paired Student’s *t*-tests was used to compare the investigated variables before and after metformin treatment within the same study population. Moreover, because data transformations must be applied very cautiously [35], the original values were recalculated using the same statistical methods, but the same results were obtained. The χ^2^ test was used to assess differences in the distribution of categorical variables. Associations between the outcome measures were analyzed using Pearson’s correlation coefficients. *p*-values less than 0.05 were deemed statistically significant. 

## 3. Results

At study entry, there were no differences between the groups in terms of age, percentages of patients with type 2 diabetes and prediabetes, smoking, BMI, systolic blood pressure, diastolic blood pressure, and daily vitamin D intake from food (Table 1). The study groups were also similar with regard to concentrations of TSH, free and total thyroid hormones, glucose, HbA_1c_, prolactin, ferritin, and osteocalcin, as well as to values of HOMA1-IR, Jostel’s index, SPINA-GT, and SPINA-GD. 25OHD concentrations were lower in group A than in the remaining groups with no differences between groups B and C (Table 1 and Table 2). The mean dose of exogenous vitamin D in group B was 82 ± 23 μg per day, and the mean duration of supplementation was 10 ± 2 months.

Only six patients (two (7.6%) in group A, three (11.5) in group B, and one (3.8%) in group C) reported adverse effects of metformin: decreased appetite, diarrhea, abdominal bloating, and a metallic taste in the mouth, which were mild and transient. The risk of adverse effects did not differ statistically between the study groups (*p* = 0.5098). The majority of participants did not experience any side effects from the drug. No patient was withdrawn from the study because of adverse effects or other reasons. Throughout the study, all participants adhered both to the treatment recommendations and to the recommendations concerning diet and physical activity. 

**Figure 2:**
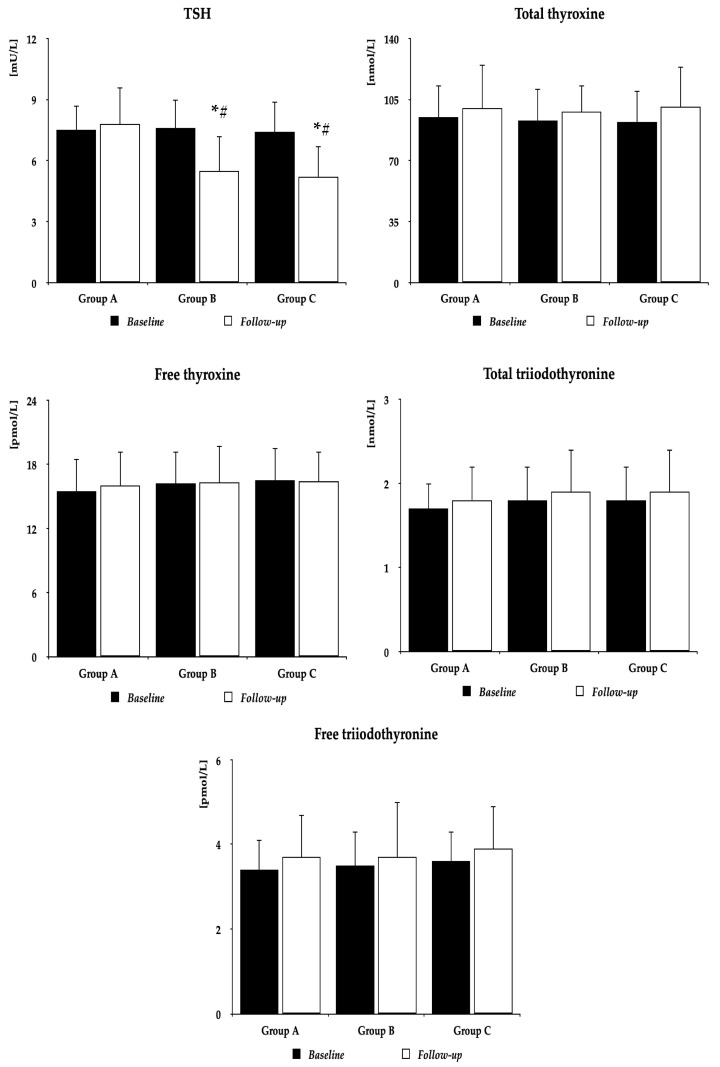
The impact of metformin on TSH and thyroid hormones. Group A: women with vitamin D deficiency or insufficiency not receiving oral preparations of exogenous vitamin D; Group B: women with normal vitamin D status receiving oral preparations of exogenous vitamin D because of previous vitamin D deficiency or insufficiency; Group C: women with normal vitamin D status not receiving preparations of exogenous vitamin D. The data are presented as the mean ± standard deviation; * *p* < 0.05 vs. baseline value, ^#^ *p* < 0.05 vs. group A. Abbreviation: TSH—thyroid-stimulating hormone.

Metformin did not affect BMI (group A: 27.6 ± 4.4 kg/m^2^ vs. 26.8 ± 4.2 kg/m^2^ (*p* = 0.5055), group B: 26.9 ± 4.3 kg/m^2^ vs. 25.8 ± 4.0 kg/m^2^ (*p* = 0.3441), group C: 26.5 ± 4.1 kg/m^2^ vs. 25.3 ± 4.1 kg/m^2^ (*p* = 0.2964)), and follow-up BMI did not differ between the study populations. Irrespective of the group, metformin decreased plasma glucose, HOMA1-IR, and HbA_1c_. In groups B and C, but not in group A, the drug also reduced TSH concentrations and Jostel’s index. There were no differences between baseline and follow-up values of 25OHD, total and free thyroxine, total and free triiodothyronine, prolactin, SPINA-GT, SPINA-GD, ferritin, and osteocalcin. Follow-up values of glucose, HOMA1-IR, HbA_1c_, TSH, and Jostel’s index were higher in group A than in the remaining two groups, with no differences between group B and group C (Figure 2 and Table 2). 

The percentage changes from baseline in glucose, HOMA-IR, HbA_1c_, TSH, and Jostel’s index were more pronounced in groups B and C than in group A (Table 3).

In all study group, the impact of metformin on TSH concentration and Jostel’s index correlated positively with baseline 25OHD concentrations. Correlations between treatment-induced changes in TSH and 25OHD were moderate (group A: r = 0.47, *p* = 0.0001, group B: r = 0.44, *p* = 0.0004, group C: r = 0.44, *p* = 0.0003), while correlations between treatment-induced changes in Jostel’s index and 25OHD were weak (group A: r = 0.35, *p* = 0.0211, group B: r = 0.36, *p* = 0.0200, group C: r = 0.37, *p* = 0.0121). In groups B and C, there were also moderate positive correlations between treatment-induced changes in TSH concentrations and baseline TSH levels (group B: r = 0.49, *p* < 0.0001, group C: r = 0.47, *p* = 0.0001), as well as between treatment-induced changes in TSH concentrations and treatment-induced changes in HOMA1-IR (group B: r = 0.41, *p* = 0.0011, group C: r = 0.40, *p* = 0.0018). The remaining correlations did not reach the level of statistical significance.

## 4. Discussion

In women with subclinical hypothyroidism and normal vitamin D status, metformin reduced elevated TSH concentration, which is in line with previous findings [12,13,14,15,16,17]. This effect was moderate (by 28–30%), probably because of relatively high baseline concentrations of TSH, and the mean follow-up TSH concentrations were still elevated. Moreover, metformin treatment did not affect total and free thyroxine and triiodothyronine. These results indicate that metformin action on the hypothalamic–pituitary–thyroid axis activity in women with thyroid hypofunction and unimpaired calciferol homeostasis is limited to the impact on TSH. 

The major finding of our study was, however, that the response of TSH to metformin was absent in women with low vitamin D status. Moreover, the degree of treatment-induced reduction in TSH (and in Jostel’s index) positively correlated with 25OHD concentration. The obtained results may partially explain why metformin/calciferol combination therapy was superior to metformin alone in reduction of circulating TSH concentration in women with high-normal TSH levels [24]. In the cited study, mean baseline 25OHD concentration was in the range for vitamin D insufficiency, co-treatment duration was long, and mean follow-up 25OHD levels suggested normalization of vitamin D homeostasis. The association between the effect of metformin on TSH concentration and vitamin D homeostasis is also supported by similar relationships between calciferol status and the strength of metformin action on prolactin and gonadotropins in the case of their overproduction [22,23]. Moreover, at baseline, partially because of matching, other variables did not differ between the study groups and cannot explain the obtained findings. Between-group differences in metformin action also cannot be associated with concomitant disorders or co-treatments. The study excluded patients with co-morbidities (with the exception of type 2 diabetes or prediabetes), while metformin and in one group exogenous calciferol were the only agents administered to patients. Thus, it seems that poor TSH response to metformin in patients with hypothyroidism may be a marker of calciferol deficiency or insufficiency and justifies assessment of vitamin D status in such patients. 

Treatment-induced reduction in TSH concentration in women with normal calciferol status was accompanied by a decrease in Jostel’s index, which indicates that the decrease in TSH concentration was probably a consequence of the inhibitory effect on thyrotroph secretory function. In turn, no changes in Jostel’s index in vitamin-D-deficient and vitamin-D-insufficient women suggest unaltered thyrotroph secretory function of women with low calciferol status in response to chronic metformin treatment. Thus, the anterior pituitary seems to be the main target for metformin action on hypothalamic–pituitary–thyroid axis activity and for interactions between metformin and calciferol. The obtained results cannot be explained in a convincing way by interactions between metformin and endogenous vitamin D at the level of the thyroid gland itself and/or peripheral target organs for thyroid hormones. There were no between-group differences in baseline and follow-up values of total and free thyroxine and triiodothyronine. Moreover, similar values of SPINA-GT and SPINA-GD in both groups suggest no differences between women with low and normal calciferol status in the amount of thyroxine released at maximum glandular TSH stimulation and in the degree of peripheral conversion of thyroxine to triiodothyronine [28]. A neutral effect on thyroid hormone secretion may be partially explained by the non-autoimmune nature of hypothyroidism in the investigated populations of hypothyroid women. Lastly, local vitamin D content did not seem to determine metformin action on thyroid hormone receptors because its administration did not affect circulating levels of ferritin and osteocalcin, and their follow-up levels were similar in both groups.

Another observation worth underlining is that the impact of metformin on the hypothalamic–pituitary–thyroid axis did not differ between both groups of patients with normal vitamin D homeostasis: subjects receiving, because of previous hypovitaminosis D, oral calciferol supplementation and women naïve to oral vitamin D supplements. This finding indicates that the TSH-lowering effect of metformin is determined by vitamin content in target organs and does not seem to be associated with pharmacokinetic or pharmacodynamic interactions between metformin and exogenous calciferol. This explanation is also supported by the lack of correlations between the impact of metformin on TSH (and the remaining outcome variables) and the daily dose of calciferol contained in its supplements, daily calciferol intake from food, and the duration of calciferol supplementation. Lastly, calciferol supplementation began at least six months before the study, and during this time and over the entire study period, the dose of exogenous vitamin D was the same. Thus, our study indicates that the inhibitory effect of low vitamin D status on metformin action on hypothalamic–pituitary–thyroid axis activity is reversible and TSH response to metformin may be restored by adequate calciferol supplementation.

Although metformin action on TSH and Jostel’s index depended on calciferol homeostasis, the drug did not affect 25OHD concentrations, irrespective of baseline calciferol status and of whether the patients were concomitantly treated with oral vitamin D supplements. A neutral effect of metformin on 25OHD was reported previously in post hoc analysis of a randomized placebo-controlled study that included both men and women with type 2 diabetes [36]. Thus, metformin does not affect vitamin D absorption and metabolism. A much more probable explanation is interaction at the level of 5′-monophosphate-activated protein kinase (AMPK). Activation of this enzyme is one of the most important mechanisms explaining metabolic effects of metformin [37]. Moreover, thyrotrophs are, besides lactotrophs, characterized by the highest expression of this enzyme in the pituitary gland [38]. Lastly, vitamin D was reported to enhance the stimulatory effect of metformin on AMPK activity, potentiating its nephroprotective action and the inhibitory effect on growth of neoplasmatic (colorectal and prostate cancer) cells [39,40,41]. According to the alternative explanation, metformin and vitamin D may act synergistically on the tuberoinfundibular pathway, one of the major dopamine pathways in the brain [42]. In line with this explanation, metformin was found to stimulate dopaminergic transmission in the hypothalamus [43], while vitamin D signaling was found to be implicated in the development of the tuberoinfundibular pathway [44].

In line with previous observations [22,23], the impact of metformin on all assessed aspects of glucose homeostasis (fasting glucose, HOMA1-IR, and HbA_1c_) was less pronounced in women with vitamin D deficiency or insufficiency. This finding is another argument supporting routine assessment of 25OHD concentration in individuals with type 2 diabetes or prediabetes poorly responding to metformin treatment and for obligatory calciferol supplementation in poor responders with low vitamin D status. Interestingly, the impact of treatment on TSH concentration moderately positively correlated with the improvement in HOMA1-IR, suggesting that metformin actions on glucose homeostasis and on hypothalamic–pituitary–thyroid axis activity are interrelated. Because the study excluded subjects with hypothyroidism of autoimmune origin, weak metabolic effects of metformin in women with low vitamin D status are likely unrelated to the impact of inflammatory mediators. It seems, however, that they may be explained by both mentioned mechanisms of interaction between metformin and calciferol. AMPK was evidenced to affect the major pathways regulating glucose homeostasis: gluconeogenesis, glycolysis, and glycogenolysis [45]. In turn, central and peripheral dopamine systems modulate glucose and energy homeostasis, improving metabolic outcome [46]. Thus, low tissue content of calciferol may impair synergistic interaction between metformin and vitamin D at the level of one or both mechanisms. 

There are some study limitations to bear in mind. Because of the study design, the findings could have been influenced by bias associated with selection of the study population and/or with confounding. The small study population, though exceeding the required sample size, made drawing strong conclusions difficult. Considering previous data [47,48], the participants seem to have been characterized by adequate iodine and inadequate selenium intake. Consequently, the association between metformin action on hypothalamic–pituitary–thyroid axis activity and vitamin D status may not be the same in iodine-deficient and/or selenium-replete subjects. The association may be also different in children, men, and postmenopausal women, all of whom were not enrolled in the study. There is a possibility that the study results could have been influenced by the regression-toward-the-mean phenomenon [49]. Lastly, because 25OHD concentrations were always below 150 nmol/L, it would be interesting to investigate pituitary effects of metformin in patients with 25OHD concentrations exceeding this threshold value.

In conclusion, long-term high-dose metformin treatment reduced TSH concentration and Jostel’s index in young women with subclinical hypothyroidism only if they had normal vitamin D status, which suggests interaction between metformin and calciferol at the level of pituitary thyrotropes. Between-group differences in response to metformin were accompanied by similar differences in metabolic effects of this drug. It seems, however, that the full hormonal and metabolic response to metformin may be restored in women with vitamin D deficiency and insufficiency after normalization of vitamin D status. Because of novelty, potential clinical relevance, and study limitations, the obtained results should be confirmed in further larger-scale prospective studies with a more diverse study population. It would also be interesting to carry out a study that allows identification of molecular and cellular mechanisms of interaction between metformin and vitamin D.

## Figures and Tables

**Table 1:** Baseline characteristics of the study groups and baseline levels of TSH and thyroid hormones in the participants.

Variable	Group A	Group B	Group C
**Number** (n)	26	26	26
**Age** (years)	35 ± 7	35 ± 8	36 ± 8
**Type 2 diabetes** (%)/**Prediabetes** (%)	38/62	42/58	38/62
**Smokers** (%)/**Number of cigarettes a day** (n)/**Duration of smoking** (years)	46/10 ± 6/14 ± 6	38/11 ± 6/13 ± 6	42/12 ± 6/15 ± 8
**BMI** (kg/m^2^)	27.6 ± 4.4	26.9 ± 4.3	26.5 ± 4.1
**Systolic blood pressure** (mmHg)	125 ± 18	123 ± 15	122 ± 17
**Diastolic blood pressure** (mmHg)	83 ± 6	82 ± 6	82 ± 5
**Vitamin D intake from food** (μg per day)	9.8 ± 3.4	10.2 ± 4.0	11.2 ± 4.3
**TSH** (mU/L)	7.5 ± 1.2	7.6 ± 1.4	7.4 ± 1.5
**Total thyroxine** (nmol/L)	95 ± 18	93 ± 20	92 ± 16
**Free thyroxine** (pmol/L)	15.5 ± 3.0	16.2 ± 2.9	16.5 ± 3.4
**Total triiodothyronine** (nmol/L)	1.7 ± 0.3	1.8 ± 0.4	1.7 ± 0.4
**Free triiodothyronine** (pmol/L)	3.4 ± 0.7	3.5 ± 0.8	3.6 ± 0.7

Group A: women with vitamin D deficiency or insufficiency not receiving oral preparations of exogenous vitamin D; Group B: women with normal vitamin D status receiving oral preparations of exogenous vitamin D because of previous vitamin D deficiency or insufficiency; Group C: women with normal vitamin D status not receiving preparations of exogenous vitamin D. Except for the percentages of smokers, patients with type 2 diabetes, and individuals with prediabetes, the data are presented as the mean ± standard deviation. Abbreviations: BMI—body mass index; TSH—thyroid-stimulating hormone.

**Table 2:** The impact of metformin treatment on 25-hydroxyvitamin D, glucose homeostasis, prolactin, structure parameters of thyroid homeostasis, and markers of thyroid hormone action.

Variable	Group A	Group B	Group C
**25OHD** (nmol/L)			
Baseline	56.5 ± 14.6	112.4 ± 20.1 *	117.0 ± 18.9 *
Follow-up	57.3 ± 15.0	114.8 ± 22.4 *	120.1 ± 21.7 *
**Glucose** (mg/dL)			
Baseline	118 ± 10	120 ± 13	117 ± 12
Follow-up	108 ± 11 *	100 ± 10 *^#^	98 ± 12 *^#^
**HOMA1-IR**			
Baseline	4.4 ± 1.4	4.6 ± 1.6	4.2 ± 1.7
Follow-up	3.4 ± 1.0 *	2.3 ± 0.8 *^#^	2.1 ± 0.6 *^#^
**HbA_1c_** (%)			
Baseline	6.6 ± 0.5	6.7 ± 0.5	6.6 ± 0.4
Follow-up	6.2 ± 0.4 *	5.6 ± 0.5 *^#^	5.5 ± 0.6 *^#^
**Prolactin** (ng/mL)			
Baseline	22.3 ± 6.8	24.4 ± 8.0	23.1 ± 8.5
Follow-up	20.6 ± 8.0	20.8 ± 7.8	20.1 ± 7.5
**Jostel’s index**			
Baseline	4.1 ± 0.2	4.2 ± 0.3	4.2 ± 0.2
Follow-up	4.2 ± 0.3	3.9 ± 0.2 *^#^	3.9 ± 0.2 *^#^
**SPINA-GT** (pmol/s)			
Baseline	1.61 ± 0.38	1.67 ± 0.40	1.72 ± 0.37
Follow-up	1.63 ± 0.46	1.85 ± 0.42	1.90 ± 0.43
**SPINA-GD** (nmol/s)			
Baseline	20.28 ± 3.56	20.00 ± 3.46	20.17 ± 3.98
Follow-up	21.38 ± 4.10	20.98 ± 3.72	21.96 ± 4.22
**Ferritin** (ng/mL)			
Baseline	42 ± 25	47 ± 30	50 ± 30
Follow-up	50 ± 28	60 ± 38	61 ± 35
**Osteocalcin** (ng/mL)			
Baseline	15.8 ± 6.2	16.2 ± 8.2	14.8 ± 5.1
Follow-up	17.4 ± 8.0	18.1 ± 7.8	17.0 ± 6.8

Group A: women with vitamin D deficiency or insufficiency not receiving oral preparations of exogenous vitamin D; Group B: women with normal vitamin D status receiving oral preparations of exogenous vitamin D because of previous vitamin D deficiency or insufficiency; Group C: women with normal vitamin D status not receiving preparations of exogenous vitamin D. The data are presented as the mean ± standard deviation; * *p* < 0.05 vs. baseline value, ^#^ *p* < 0.05 vs. group A; Abbreviations: HbA_1c_—glycated hemoglobin, HOMA1-IR—the homeostatic model assessment 1 of insulin resistance ratio; SPINA—structure parameter inference approach; 25OHD—25-hydroxyvitamin D.

**Table 3:** Percentage changes from baseline in the investigated variables during metformin treatment.

Variable	Group A	Group B	Group C
Δ **25OHD**	1 ± 5	2 ± 8	3 ± 7
Δ **Glucose**	−8 ± 5	−17 ± 4 *	−16 ± 5 *
Δ **HOMA1-IR**	−23 ± 18	−50 ± 20 *	−50 ± 25 *
Δ **HbA_1c_**	−6 ± 2	−16 ± 4 *	−17 ± 4 *
Δ **TSH**	4 ± 8	−28 ± 10 *	−30 ± 11 *
Δ **Total thyroxine**	5 ± 12	5 ± 11	10 ± 16
Δ **Free thyroxine**	3 ± 10	1 ± 8	−1 ± 5
Δ **Total triiodothyronine**	6 ± 10	6 ± 8	12 ± 14
Δ **Free triiodothyronine**	9 ± 12	6 ± 8	9 ± 11
Δ **Prolactin**	−8 ± 10	−15 ± 17	−13 ± 12
Δ **Jostel’s index**	2 ± 4	−7 ± 5 *	−7 ± 6 *
Δ **SPINA-GT**	2 ± 12	10 ± 19	10 ± 20
Δ **SPINA-GD**	5 ± 11	5 ± 12	9 ± 10
Δ **Ferritin**	19 ± 20	28 ± 23	22 ± 20
Δ **Osteocalcin**	10 ± 20	12 ± 18	15 ± 16

Group A: women with vitamin D deficiency or insufficiency not receiving oral preparations of exogenous vitamin D; Group B: women with normal vitamin D status receiving oral preparations of exogenous vitamin D because of previous vitamin D deficiency or insufficiency; Group C: women with normal vitamin D status not receiving preparations of exogenous vitamin D. The data are presented as the mean ± standard deviation; * *p* < 0.05 vs. group A; Abbreviations: HbA_1c_—glycated hemoglobin, HOMA1-IR—the homeostatic model assessment 1 of insulin resistance ratio; SPINA—structure parameter inference approach; TSH—thyroid-stimulating hormone; 25OHD—25-hydroxyvitamin D.

## Data Availability

The data that support the findings of this study are available from the corresponding author upon reasonable request.

## Abbreviations:

AMPK—5′-adenosine monophosphate-activated protein kinase; BMI—body mass index; HbA_1c_—glycated hemoglobin, HOMA1-IR—the homeostatic model assessment 1 of insulin resistance ratio; SPINA—structure parameter inference approach; TSH—thyroid-stimulating hormone; 25OHD—25-hydroxyvitamin D.
